# Complementary expression of calcium binding proteins delineates the functional organization of the locomotor network

**DOI:** 10.1007/s00429-018-1622-4

**Published:** 2018-02-08

**Authors:** Eva M. Berg, Maria Bertuzzi, Konstantinos Ampatzis

**Affiliations:** Department of Neuroscience, Karolinska Institutet, 171 77 Stockholm, Sweden

**Keywords:** Calbindin, Calretinin, Parvalbumin, Spinal cord, Zebrafish

## Abstract

Neuronal networks in the spinal cord generate and execute all locomotor-related movements by transforming descending signals from supraspinal areas into appropriate rhythmic activity patterns. In these spinal networks, neurons that arise from the same progenitor domain share similar distribution patterns, neurotransmitter phenotypes, morphological and electrophysiological features. However, subgroups of them participate in different functionally distinct microcircuits to produce locomotion at different speeds and of different modalities. To better understand the nature of this network complexity, here we characterized the distribution of parvalbumin (PV), calbindin D-28 k (CB) and calretinin (CR) which are regulators of intracellular calcium levels and can serve as anatomical markers for morphologically and potential functionally distinct neuronal subpopulations. We observed wide expression of CBPs in the adult zebrafish, in several spinal and reticulospinal neuronal populations with a diverse neurotransmitter phenotype. We also found that several spinal motoneurons express CR and PV. However, only the motoneuron pools that are responsible for generation of fast locomotion were CR-positive. CR can thus be used as a marker for fast motoneurons and might potentially label the fast locomotor module. Moreover, CB was mainly observed in the neuronal progenitor cells that are distributed around the central canal. Thus, our results suggest that during development the spinal neurons utilize CB and as the neurons mature and establish a neurotransmitter phenotype they use CR or/and PV. The detailed characterization of CBPs expression, in the spinal cord and brainstem neurons, is a crucial step toward a better understanding of the development and functionality of neuronal locomotor networks.

## Introduction

A plethora of neuronal functions were attributed to calcium binding proteins (CBPs), including neuronal excitability, neurotransmitter release, and excitotoxicity (Baimbridge et al. 1992; Andressen et al. 1993; Schwaller et al. 2002). Calretinin (CR), calbindin D28-k (CB) and parvalbumin (PV) are three major EF-hand CBPs that play significant roles in the regulation of intracellular Ca^2+^ homeostasis by buffering and transporting Ca^2+^ (Blaustein 1988; Heizman and Braun 1992; Andressen et al. 1993; Chard et al. 1993; Berridge et al. 2000). Although the precise physiological function of PV, CB and CR is still not fully understood, each of them individually or in combinations has been demonstrated to be a valuable marker of separate neuron populations in the vertebrate central nervous system (Arai et al. 1991; Baimbridge et al. 1992; Resibois and Rogers 1992; Andressen et al. 1993; Kress et al. 2015) including the spinal cord (Fournet et al. 1986; Antal et al. 1990, 1991; Celio 1990; Ince et al. 1993; Ren et al. 1993; Megías et al. 2003; Anelli and Heckman 2005; Morona et al. 2006a, b). The presence of CBPs in different neuronal populations, such as cholinergic, GABAergic, glutamatergic and nitrinergic (Baimbridge et al. 1992), supports the notion that CBPs are not associated to any neurochemical specificity of neurons. However, in numerous studied areas within the nervous system, they are localized in nearly non-overlapping cell assemblies. This segregated distribution pattern allows the identification of subgroups within nuclei that represent discrete neuronal micro-circuits, which are not cyto-architecturally separated (Andressen et al. 1993), but may perform different functions.

In all vertebrates, locomotion relies on the activation of central pattern-generating networks located within the spinal cord (Grillner 2003, 2006; Goulding 2009; Kiehn 2006; Grillner and Jessell 2009). These defined spinal networks transform descending supraspinal signals to generate movements with diverse speeds and of different modalities (Grillner and Jessell 2009; Esposito et al. 2014; Kiehn 2016), and they are formed by a highly heterogeneous population of neurons. An important step towards understanding the principles that govern the organization and functionality of spinal locomotor circuits is to determine the identity of the different spinal neuron populations. Numerous types of neurons have been already described based on their developmental origin, and morphological and electrophysiological properties in the vertebrate spinal cord (Jankowska 1992; Jessell 2000; Briscoe and Ericson 2001; Lee and Pfaff 2001; Goulding et al. 2002; Sueiro et al. 2004; Grillner 2006; Kiehn 2006; Lewis 2006; Windhorst 2007; McCrea and Rybak 2008; Mahmood et al. 2009; Berkowitz et al. 2010; Bikoff et al. 2016) including zebrafish (Bernhardt et al. 1990; Hale et al. 2001; Drapeau et al. 2002; McLean and Fetcho 2004; Higashijima et al. 2004a, b, c; Kimura et al. 2008; Satou et al. 2009, 2012; Bradley et al. 2010; Ampatzis et al. 2013; Ferg et al. 2014; Menelaou et al. 2014; Böhm et al. 2016). The continuous adjustment of locomotor speed relies on the precise recruitment of distinct spinal interneurons and motoneurons. While neurons that belong to the same populations in zebrafish spinal cord, as they arise from the same progenitor pool, share similar morphological and electrophysiological properties and release the same neurotransmitter, they are functionally distinct, in terms of their recruitment plan (McLean et al. 2007; Gabriel et al. 2011; Ausborn et al. 2012; Ampatzis et al. 2013; Kishore et al. 2014; Menelaou et al. 2014; Björnfors and El Manira 2016). To this end, studies in adult zebrafish have shown that the generation of locomotion at different speeds relies on sequential activation of functionally distinct subpopulations (slow, intermediate and fast) of interneurons (Ausborn et al. 2012; Ampatzis et al. 2014; Björnfors and El Manira 2016) and motoneurons (Gabriel et al. 2011; Ampatzis et al. 2013). To understand further the nature of this functional complexity of spinal circuitry organization, we characterized the distribution pattern of CBPs, regulators of intracellular calcium that serve as valuable anatomical markers for morphologically and potential functionally distinct neuronal subpopulations. In the present work, we provide a detailed description of the distribution pattern of calretinin (CR), calbindin D28-k (CB) and parvalbumin (PV) containing neurons to determine the relationship between the type of calcium binding proteins present in adult zebrafish spinal cord and brain descending neurons and the accompanying function.

We first show that CR and PV containing neurons were co-distributed and occasionally co-localized in motoneurons and interneurons with a diverse neurotransmitter phenotype in the adult zebrafish spinal cord. In contrast, CB immunoreactivity was observed in neuronal progenitor cells that were distributed around the central canal. We then show that the calcium binding protein CR is highly expressed in fast and in few intermediate motoneurons but not in slow motoneurons. Our results suggest that during development the spinal cord neurons utilize CB as an intracellular buffer protein and as they mature and establish a neurotransmitter phenotype they use CR or/and PV. Moreover, our findings propose that motoneurons which are involved in fast modalities of locomotion, such as fast swimming and escape, require an additional regulator for their intracellular calcium, and therefore CR can be potentially used as an anatomical marker for the fast locomotor system. We believe that such comprehensive analysis is necessary and potentially highly valuable as a framework for ongoing and future studies in the spinal neuronal networks controlling generation of locomotion at different speeds and modalities.

## Materials and methods

### Experimental animals

All animals were raised and kept in the core facility at the Karolinska Institute according to established procedures. Adult zebrafish (*Danio rerio; n* = 190; 10–12 weeks old; length, 16–19 mm; weight, 0.03–0.05 g) wild type (AB/Tübingen) of either sex where used in this study.

### Motoneuron and descending neuron labeling

Zebrafish (*n* = 48) of either sex were anesthetized in 0.03% tricaine methane sulfonate (MS-222, Sigma-Aldrich) and placed, lying lateral side up, onto a wet paper tissue inside a petri dish. Retrograde labeling of axial motoneurons was performed by dye injections of tetramethylrhodamine-dextran (3000 MW; ThermoFisher, D3307) into specific muscle fiber types (slow, intermediate or fast), which in the adult zebrafish are arranged in an anatomically segregated manner, as described before in detail (Ampatzis et al. 2013). In addition, retrograde labeling of all motoneurons was performed by applying similar procedures to spinal cord ventral roots. To label the neurons descending from the brain to the spinal cord, dye was injected into the spinal cord at approximately the level of the 8-10th vertebra. Afterwards, animals were kept for at least 24 h to allow for retrograde transport of the tracer.

### Immunohistochemistry

All animals were deeply anesthetized with 0.1% MS-222. We then dissected the spinal cords and/or the brains and fixed them in 4% paraformaldehyde (PFA) in phosphate buffer saline (PBS) (0.01M; pH 7.4) at 4 °C for 2–14 h. We performed immunolabeling in both whole mount spinal cords and in cryosections. For cryosections, the tissue was removed carefully and cryoprotected overnight in 30% (w/v) sucrose in PBS at 4 °C, embedded in OCT Cryomount (Histolab), rapidly frozen in dry-ice-cooled isopentane (2-methylbutane; Sigma) at approximately − 35 °C, and stored at − 80 °C until use. Transverse coronal plane cryosections (thickness 25 µm) of the tissue were collected and processed for immunohistochemistry. In all cases, the tissue was washed three times for 5 min in PBS. Nonspecific protein binding sites were blocked with 4% normal donkey serum with 1% bovine serum albumin (BSA; Sigma) and 1% Triton X-100 (Sigma) in PBS for 30 min at room temperature (RT). Primary antibodies (Table 1) were diluted in 1% of blocking solution and applied for 24–90 h at 4 °C. After thorough buffer rinses, the tissue was then incubated with the appropriate secondary antibodies (Table 1) diluted 1:500 in 1% Triton X-100 (Sigma) in PBS overnight at 4 °C. Finally, the tissue was thoroughly rinsed in PBS and cover-slipped with fluorescent hard medium (VectorLabs; H-1400).

**Table 1:** Antibodies used

Antigen	Host	Source	Code	Dilution
Primary				
PV	Mouse	Swant	235	1:3000
PV	Rabbit	Swant	PV27	1:3000
CR	Rabbit	Swant	CR7697	1:500
CR	Mouse	Swant	6B3	1:1000
CB D-28 k	Mouse	Swant	300	1:2000
CB D-28 k	Rabbit	Millipore	AB1778	1:200
ChAT	Goat	Chemicon	AB144P	1:150
Islet1	Mouse	DSHB	40.2D6	1:100
GABA	Rabbit	Sigma	A2052	1:2000
Glycine	Rat	ImmunoSolution	IG1002	1:1000
Glutamate	Rabbit	Sigma	G6642	1:4000
Serotonin	Rabbit	Sigma	S5545	1:4000
Sox-2	Goat	R&D Systems	AF2018	1:500
Elav3 + 4 (HuC/D)	Rabbit	GeneTex	GTX128365	1:500
Secondary				
Goat IgG-568	Donkey	ThermoFisher	A-11057	1:500
Mouse IgG-647	Donkey	ThermoFisher	A-31571	1:500
Mouse IgG-568	Goat	ThermoFisher	A-11004	1:500
Mouse IgG-488	Donkey	ThermoFisher	A-21202	1:500
Rat IgG-550	Donkey	ThermoFisher	SA5-10027	1:500
Rabbit IgG-488	Donkey	ThermoFisher	A-21206	1:500
Rabbit IgG-568	Donkey	ThermoFisher	A-10042	1:500

*CB* calbindin D-28 k, *CR* calretinin, *PV* parvalbumin, *ChAT* choline-acetyltransferase, *GABA* γ-Aminobutyric acid

The antibodies used in this study have been widely used in zebrafish before and have been described to reliably identify neurotransmitter phenotypes (anti-ChAT: Clemente et al. 2004; Mueller et al. 2004, 2006; Reimer et al. 2008; Moly et al. 2014; Ohnmacht et al. 2016; anti-GABA; Higashijima et al. 2004a; Montgomery et al. 2016; Djenoune et al. 2017; anti-Glycine; anti-Serotonin; Kuscha et al. 2012; McPherson et al. 2016). To further evaluate the antibody specificity, adjacent sections or additional whole mount spinal cords were used in the absence of the first or second antibody. In all cases, no residual immunolabeling was detected. Furthermore, to assess the specificity of antibodies against the selected neurotransmitters (GABA, glutamate, glycine and serotonin), we pre-incubated the neurotransmitter antibodies used in this study with their corresponding antigen for 1 h at RT (100–400 µΜ) GABA (A2129, Sigma-Aldrich), glutamate (G3291, Sigma-Aldrich), glycine (G6761, Sigma-Aldrich), and serotonin (14927, Sigma-Aldrich) which eliminated any immunoreactivity. In addition, we performed similar experiments in transgenic zebrafish lines (*Gad1b:GFP, Vglut2:GFP, Glyt2:GFP* and *Tph2:GFP*), in which the majority of the respective neurons express green fluorescent protein (GFP). In all cases, most of the GFP^+^ neurons were also immunolabeled with antibodies (data not shown) suggesting the specificity of our immunodetection.

### Analysis

Imaging was carried out in a laser scanning confocal microscope (LSM 510 Meta, Zeiss). Cell counting was performed in segment 15 of the adult zebrafish spinal cord (in whole mount preparations), or in non-overlapping fields of spinal cord sections, between 14 and 16 spinal cord segments. The relative position of the somata of neurons within the spinal cord was calculated in whole mount preparations, using the lateral, dorsal, and ventral edges of the spinal cord as well as the central canal as landmarks. The relative position was calculated using ImageJ. Examination of the descending neurons was performed from a series of coronal brain sections, throughout the brain. Cells of each analyzed brain area were counted in a section that sampled the area well. The nomenclature used for the brain areas of descending neurons was based on the topological zebrafish brain atlas (Wullimann et al. 1996). All figures and graphs were prepared with Adobe Photoshop and Adobe Illustrator (Adobe Systems Inc., San Jose, CA, USA). Digital modifications of the images (changes in brightness and contrast) were minimal to not affect the biological information. All images from double-labeling immunofluoresence experiments were *post hoc* converted to magenta-green to make this work more accessible to red-green color-blind readers.

### Statistics

The significance of differences between the means in experimental animal groups for the detection of CBPs was analyzed using One-way ANOVA followed by post hoc Tukey, using Prism (GraphPad Software Inc.). Differences were considered to be significant if *p* < 0.05. Data presented here are given as mean ± SEM.

## Results

### Distribution pattern of calcium binding proteins in the adult zebrafish spinal cord

**Fig. 1:**
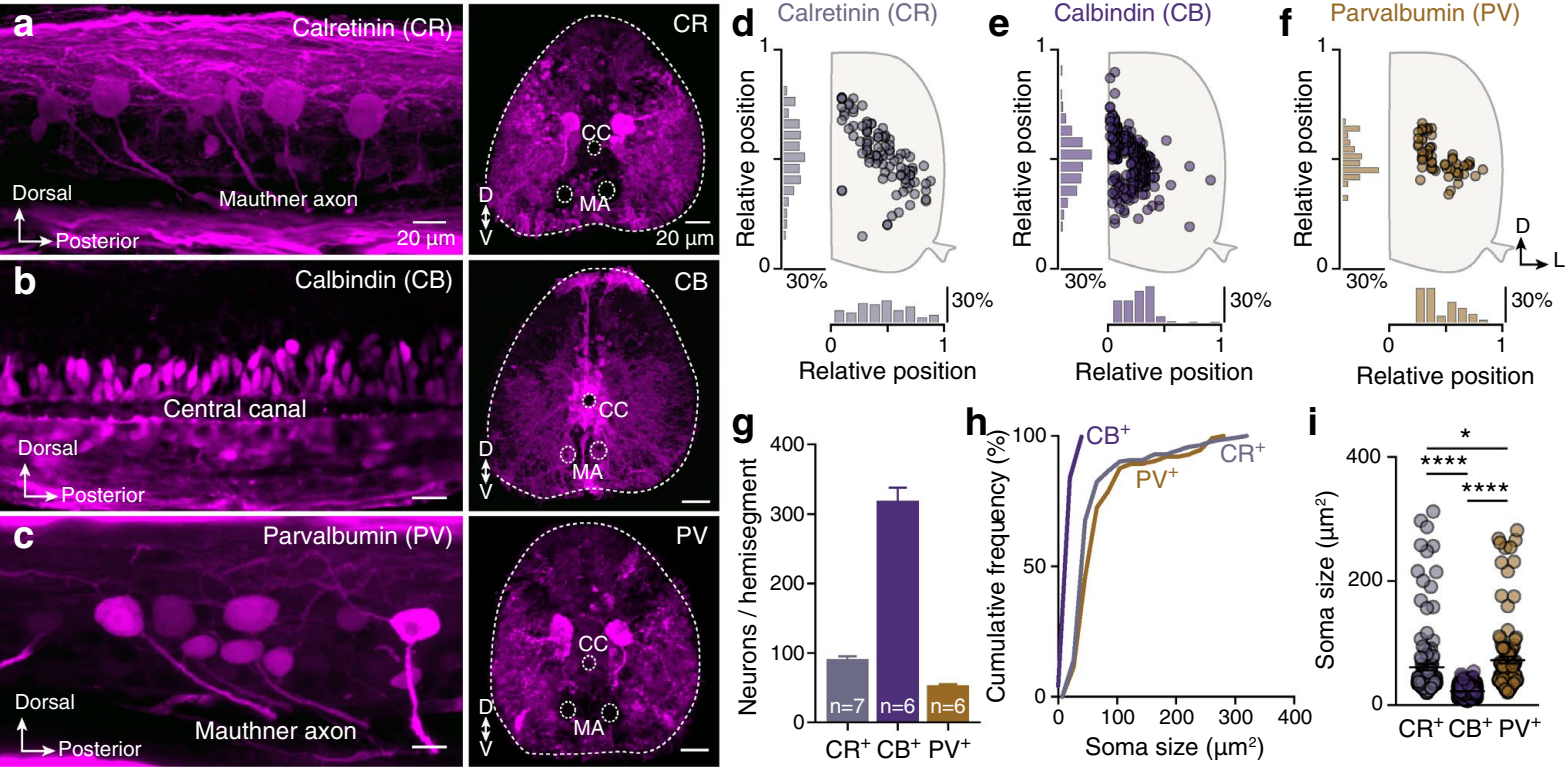
Overview of calretinin (CR), calbindin (CB) and parvalbumin (PV) expression in the adult zebrafish spinal cord. **a**–**c** Expression of CBPs in whole mount and transverse sections of the adult zebrafish spinal cord (segment 15). **d**–**f** Representative setting positions of the CR, CB and PV positive neurons in spinal hemisegments 15. **g** Number of CR, CB and PV positive neurons per adult zebrafish spinal hemisegment. **h, i** Cumulative frequency (h) and average of soma sizes for CR, CB and PV positive neurons. The soma size between CR, CB and PV containing neurons is different (one-way ANOVA: *F*_(2, 645)_ = 110, *p* < 0.0001). Data are presented as mean ± SEM; asterisks indicate statistical significance. *p < 0.05; ****p < 0.0001

To determine the expression pattern of the three major CBPs (CR, CB and PV), we analyzed their immunoreactivity in the whole hemisegment that corresponds to segment 15 of the adult zebrafish spinal cord. The detailed distribution analysis revealed that CR^+^ and PV^+^ neurons are co-distributed throughout the motor column, from the most ventrolateral to middle part (Fig. 1a, c, d, f). In addition to the neuronal somata staining observed, profuse fiber labeling was also present in the neuropil (Fig. 1a, c) where the spinal motoneuron and interneuron dendrites are extending. Immunoreactivity for both CR and PV was observed in various body sized neurons (CR^+^: 61.57 ± 4.85 µm^2^, *n* = 3 zebrafish; PV^+^: 72.87 ± 5.68 µm^2^, *n* = 3 zebrafish; Fig. 1h,i). Analysis of the complete number of CB^+^ neurons in zebrafish spinal cord hemisegment revealed a neuronal population significantly larger (136.2 ± 3.19 neurons/hemisegment, *n* = 7 zebrafish, Fig. 1g) than that observed for CR and PV (CR^+^: 56.57 ± 2.94 neurons/hemisegment, *n* = 6 zebrafish; PV^+^: 43.5 ± 0.99 neurons/hemisegment, *n* = 6 zebrafish, Fig. 1g). Numerous small sized CB^+^ neurons (22.51 ± 0.39 µm^2^, *n* = 3 zebrafish, Fig. 1i) were primarily present in the middle part of the spinal cord, in close apposition to the central canal. Dorsal spinal regions practically lacked the selected CBP^+^ cells. Overall, these observations were extremely consistent from animal to animal.

**Fig. 2:**
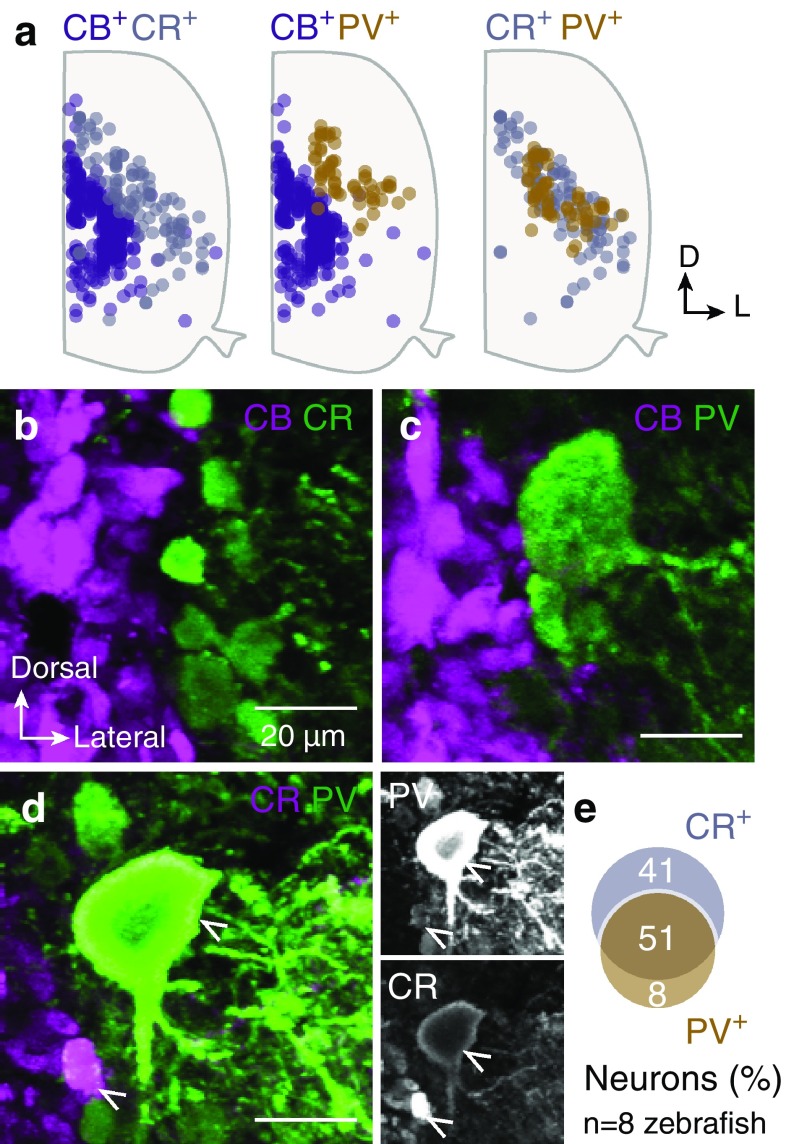
Co-distribution and co-localization of the CBPs positive neurons. **a** Superimposed positions of the CR, CB and PV positive neurons in the spinal cord. **b**–**d** Double immunofluorescent images between the three studied CBPs (CR, CB and PV). Many double expressing neurons observed only between CR and PV (51%; arrowheads in **d**). **e**  Percentage of co-expression of CR, CB or PV in neurons. Only a small population of PV^+^ neurons does not express CR (8%; **e**). Single channel views of the respective framed box

The wide co-distribution of CR^+^ and PV^+^ neurons in the same area of the adult zebrafish spinal cord and their expression in almost similar sized neurons (Figs. 1i, 2a) strongly suggested the possibility of co-localization of both CBPs in the same population of neurons. Thus, to test and estimate the proportion of co-localization of the different CBPs in neurons, double-labeling experiments were performed. There was no co-expression of CB with either of the other calcium buffering proteins (CR or PV, *n* = 6 zebrafish, Fig. 2a–c). In contrast, the majority of PV^+^ neurons were found to express also CR (CR^+^PV^+^: 51%, *n* = 8 zebrafish, Fig. 2d, e). In addition, a population of CR^+^PV^−^ neurons (41%) and a small population of CR^−^PV^+^ neurons (8%) were also detected (Fig. 2e). Overall, these data clearly show distinct cytoarchitectural distribution patterns of CR^+^, CB^+^ and PV^+^ neurons in adult zebrafish spinal cord.

### Calcium binding proteins are localized in distinct neurochemical populations

Neuronal control of movements is organized by a heterogeneous population of spinal neurons (interneurons and motoneurons) characterized by specific neurotransmitter phenotypes (Grillner 2003; Kiehn 2006; Goulding 2009). To explore the relationship between CBP expression and neurotransmitter typology of different spinal neuronal populations, we sought to establish a detailed map of CR, CB or PV immunoreactivity in GABAergic, glycinergic, glutamatergic, cholinergic and serotonergic neurons in the adult zebrafish spinal cord.

**Fig. 3:**
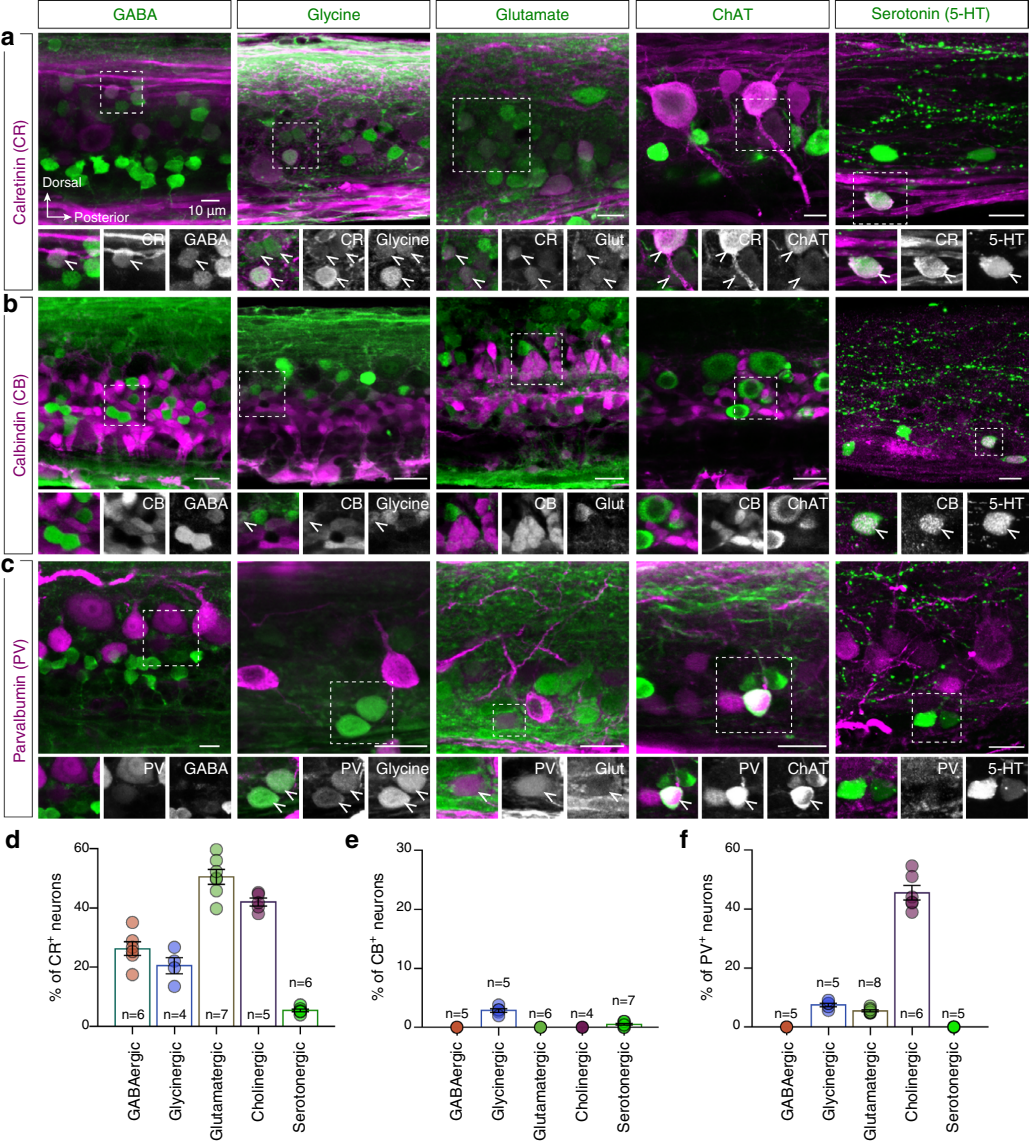
CR, CB and PV expression in neurons with identified neurotransmitter type. **a**–**c** Double immunostaining experiments for CR, CB and PV (magenta) with markers that label the GABAergic, glycinergic, glutamatergic, cholinergic and serotonergic spinal neurons (green) in whole mount adult zebrafish spinal preparations. Enlarged and single channel views are given for areas indicated by framed boxes. Arrowheads indicate double-labeled cells. **d**–**f** Quantification of the percentage of CR, CB and PV positive neurons that localized in neurons with a specific neurotransmitter phenotype

In the vertebrate nervous system, the presence of calcium binding proteins is often associated with specific neurotransmitter phenotypes (Katsumaru et al. 1988; Celio 1990; Andressen et al. 1993). However, the reason why some cell types express CR, CB, or PV and correlate to neurons with a specific neurotransmitter phenotype is not clear yet. Double immunofluorescence experiments revealed that fractions of CR containing neurons were GABAergic (26.27 ± 2.36%, *n* = 6 zebrafish), glycinergic (20.54 ± 2.75%, *n* = 4 zebrafish), glutamatergic (50.51 ± 2.47%, *n* = 7 zebrafish), cholinergic (42.04 ± 1.32%, *n* = 5 zebrafish) and serotonergic (5.44 ± 0.45%, *n* = 6 zebrafish) (Fig. 3a, d). None of the PV^+^ neurons were found to be GABAergic (*n* = 5 zebrafish) or serotonergic (*n* = 5 zebrafish). However, similar to CR immunoreactivity, 7.52 ± 0.59% (*n* = 5 zebrafish), 5.48 ± 0.58% (*n* = 8 zebrafish) and 45.51 ± 2.45% (*n* = 6 zebrafish) of PV^+^ neurons were glycinergic, glutamatergic and cholinergic neurons, respectively (Fig. 3c, f). Despite the high degree of co-distribution between CB^+^ and GABA^+^ neurons, no double-labeled cells were observed in the middle part of the spinal cord close to central canal (Fig. 3b, e). In addition, none of the CB^+^ neurons were found to express glutamate (*n* = 6 zebrafish) or ChAT (*n* = 4 zebrafish), and only few CB^+^Glycine^+^ (2.85 ± 0.29%, *n* = 5 zebrafish) and CB^+^Serotonin^+^ (0.49 ± 0.15%, *n* = 7 zebrafish) neurons were observed (Fig. 3b, e). Collectively, these data reveal that CR^+^ and PV^+^ neurons localized in a phenotypically heterogeneous population of inhibitory and excitatory interneurons and in motoneurons within the adult zebrafish spinal cord. Moreover, CB immunoreactivity was not specifically related to any particular major neurotransmitter phenotype neuronal population.

### Calretinin classifies the fast motoneuron module

**Fig. 4:**
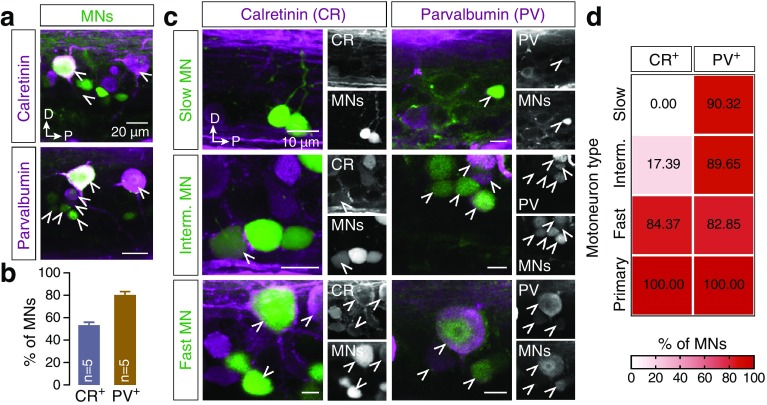
Zebrafish axial motoneurons are immunoreactive to CR and PV. **a** Representative images of the double-labeled motoneurons with CR and PV. **b** Quantification of the percentage of the motoneurons that express both CR and PV. **c** Whole mount images showing the expression of CR and PV in distinct motoneuron pools (slow, intermediate and fast). Single channel views of the respective images shown for better visualization of CR or PV positive cells. Arrowheads indicate the double-labeled cells. **d** Color coded quantification of the CR and PV expression in distinct populations of neurons. The vast majority of motoneurons contain PV. CR expression is localized mainly in the motoneurons of the fast module

Numerous CR and PV containing neurons had a cholinergic neurochemical phenotype (Fig. 3a, c, d, f), which labels the motoneurons and the cholinergic interneurons in the adult zebrafish spinal cord. To test whether either of these CBPs co-localized with motoneurons, retrograde tracer was injected into ventral roots to label the motoneurons. Indeed, several motoneurons expressed CR (53.25 ± 2.74%, *n* = 5 zebrafish, Fig. 4a, b) and the vast majority of labeled motoneurons was found to express PV (80.14 ± 3.16%, *n* = 5 zebrafish, Fig. 4a, b).

To further determine the expression pattern of CR and PV in functionally different motoneuron pools (slow, intermediate, fast), retrograde tracer was injected into the respective different muscle fiber types by taking advantage of the accessible neuromuscular configuration of the adult zebrafish (Ampatzis et al. 2013). Overall, 90.32% (28 out of 31 neurons), 89.65% (26 out of 29 neurons), 82.85% (29 out of 35 neurons) and 100% (15 out of 15 neurons) of slow, intermediate, fast and primary motoneurons, respectively, were immunoreactive for PV (Fig. 4c, d). In contrast, CR immunoreactivity was more confined to motoneurons responsible for the contraction of fast muscles. All primary motoneurons were CR^+^ (100%; 14 out of 14 neurons) as well as the vast majority of fast motoneurons (84.37%; 4 out of 23 neurons) (Fig. 4c, d). In contrast, a small fraction of intermediate motoneurons was found to express CR (17.39%; 27 out of 32 neurons) and none of the slow motoneurons contained CR (0%; 0 out of 27 neurons) (Fig. 4c, d). Our results thus suggest that CR^+^ expression can be a potential marker of the fast module of the locomotor network (Ampatzis et al. 2014).

### Calcium binding protein expression in brain neurons that descend to the spinal cord and initiate locomotion

**Fig. 5:**
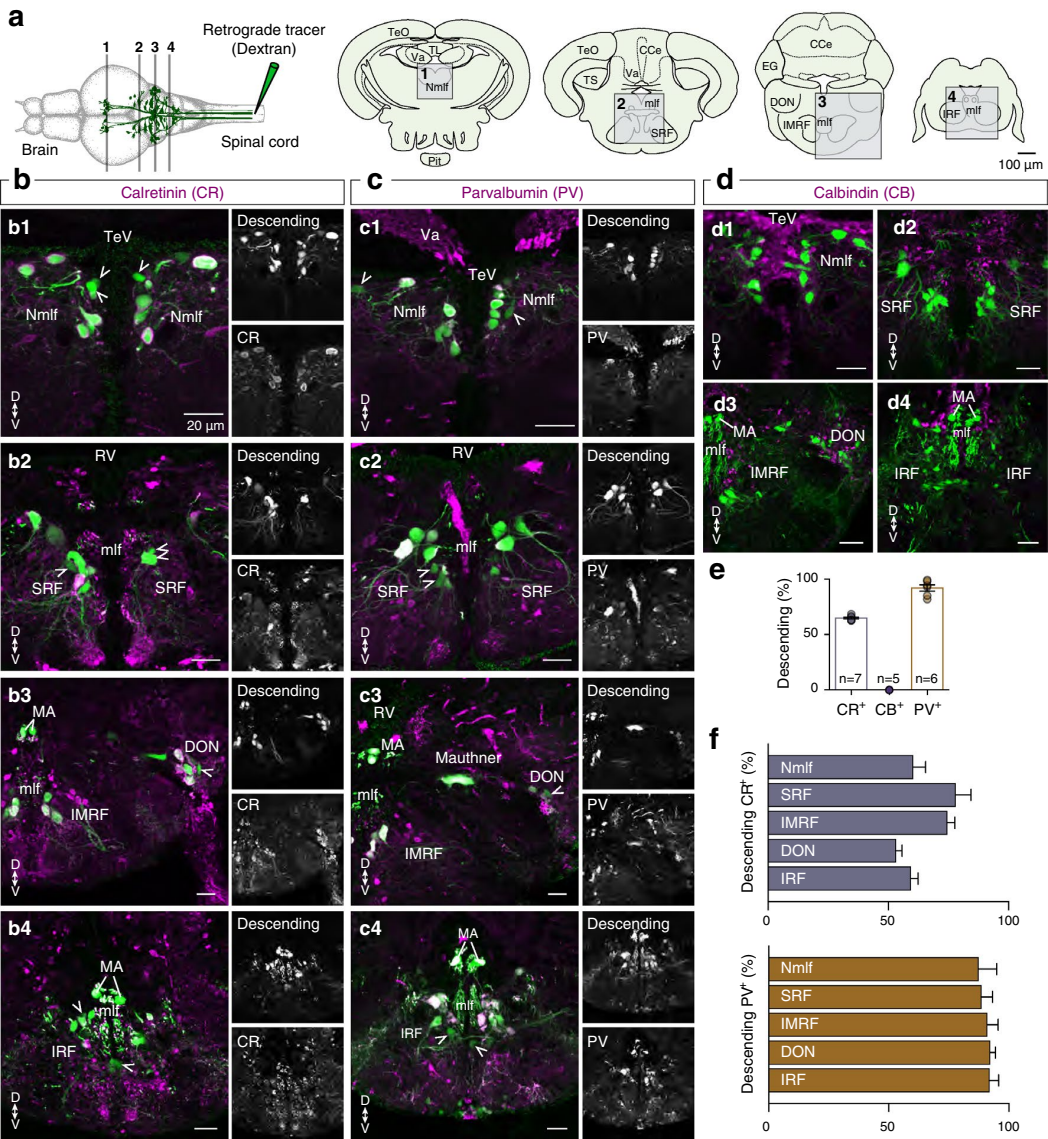
Distribution pattern of CR, CB and PV expression in reticulospinal neurons. **a** Schematic representation of the methodology used to reveal the brain neurons projecting to the spinal cord. The analyzed brain areas are indicated by boxes in coronal sections 1–4. **b**–**d** Microphotographs from coronal brain sections, showing the distribution of the brain neurons that project to the spinal cord (green) and the CR, CB and PV expression (magenta). Single channel views of the respective images shown for better visualization of the double-labeled neurons. Arrowheads indicate the double-labeled cells. **e** Percentage of the reticulospinal neurons that express CR, CB or PV, respectively. **f** Bar graph showing the percentage of brain to spinal cord projecting neurons in several brain areas that express CR or PV

Previous studies showed that the spinal locomotor circuitry of adult zebrafish is organized in three separate microcircuit modules named the slow, the intermediate and the fast (Ampatzis et al. 2014). Although spinal networks are capable and sufficient to produce all locomotion related movements (Grillner 2003, 2006; Grillner and Jessell 2009), the initiation of any motor event arises from descending signals from supraspinal areas (Grillner and Jessell 2009; Esposito et al. 2014; Kiehn 2016). To evaluate the distribution of CBPs in supraspinal neurons that innervate the spinal cord, and their potential to reveal and discriminate the possible existence of functionally segregated descending populations, a series of experiments combining tracing techniques and immunohistochemistry were conducted (Fig. 5a). The double-labeling experiments showed a wide distribution of CR^+^ or PV^+^ brain descending neurons in several brain areas (Fig. 5b, c). Retrogradely labeled brain neurons that showed CR and PV expression were observed in the nucleus of the medial longitudinal fascicle (Nmlf; PV: 87.35 ± 7.68%, CR: 60.19 ± 5.3%, Fig. 5f), superior reticular formation (SRF; PV: 88.51 ± 4.76%, CR: 77.92 ± 6.46, Fig. 5f), intermediate reticular formation (IMRF; PV: 90.94 ± 4.6%, CR: 74.31 ± 3.29%, Fig. 5f), inferior reticular formation (IRF; PV: 91.79 ± 4%, CR: 59.26 ± 3.15%, Fig. 5f), and in the descending octaval nucleus (DON; PV: 92.14 ± 2.29%, CR: 53.06 ± 2.67%, Fig. 5f). Our analysis showed that the vast majority of descending supraspinal neurons contained PV (92.08 ± 2.85%, *n* = 6 zebrafish), however, no more than 64.89 ± 0.79% (*n* = 7 zebrafish) of these neurons were found to contain CR (Fig. 5e). Additionally, our analysis showed that none of the retrogradely labeled brain neurons expressed CB (Fig. 5d). Collectively, our data suggest that CR can be a valuable marker to define potentially functionally distinct subpopulations of brain to spinal cord descending neurons.

### Calbindin defines the spinal neuronal progenitors

**Fig. 6:**
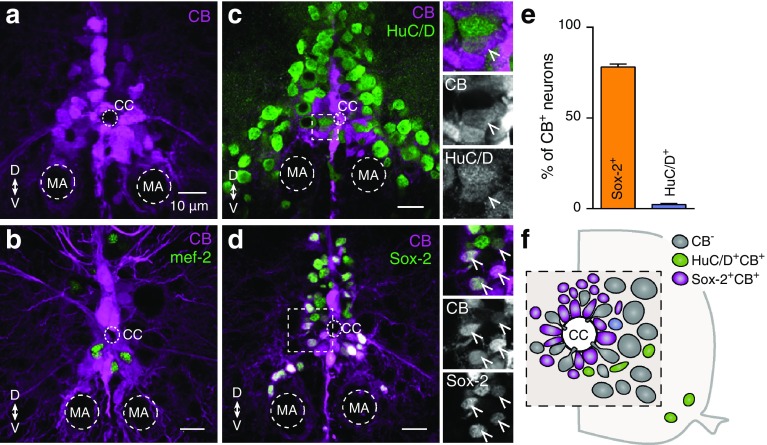
Identity of the CB positive neurons. **a** Distribution pattern of CB^+^ expression around the central canal in the adult zebrafish spinal cord. **b** None of the CB expressing cells (magenta) co-localized with the early neuronal differentiated marker mef-2 (green). **c**–**d** A small number (2.32%) of the differentiated and mature neurons (HuC/D^+^, green) contain also CB (magenta). In addition, the vast majority (78.13%) of CB containing cells (magenta) is progenitor cells / stem cells (Sox-2^+^, green). Single channel views shown for areas are indicated by framed rectangles. Arrowheads indicate double labeled cells. **e** Quantification of CB positive cells that co-express Sox-2^+^ or HuC/D^+^. **f** Schematic representation of the central canal area in the adult zebrafish spinal cord showing that most of the CB^+^ cells are progenitors / stem cells

CB immunoreactivity was not specifically related with any particular major neurochemical phenotype (Fig. 3e). In addition, CB^+^ cells were detected mainly around the central canal (Fig. 6a), the proliferation niche of the spinal cord (Grandel et al. 2006; Kaslin et al. 2008; Hui et al. 2015). This raises the possibility that CB might label undifferentiated new born cells. To test this hypothesis, we performed a series of experiments to identify the nature of the CB^+^ cells. We observed that none of the newly differentiated and migrated neurons (marked with mef-2^+^) were CB^+^ (Fig. 6b). Moreover, only a small fraction of CB^+^ cells were found to express HuC/D (2.32 ± 0.561%, *n* = 5; Fig. 6c, e, f) a marker for postmitotic neurons. Finally, we observed that the vast majority (78.13 ± 1.541%, *n* = 35; Fig. 6d, e, f) of CB^+^ cells co-expressed Sox-2, a marker for neuronal progenitor cells and stem cells (Ferri et al. 2004; Episkopou 2005; Wegner and Stolt 2005; Takahashi and Yamanaka 2006). Taken together, these findings demonstrate that CB can serve as a valuable anatomical marker to identify the mitotically active cells that are able to generate neurons in the spinal cord.

## Discussion

**Fig. 7:**
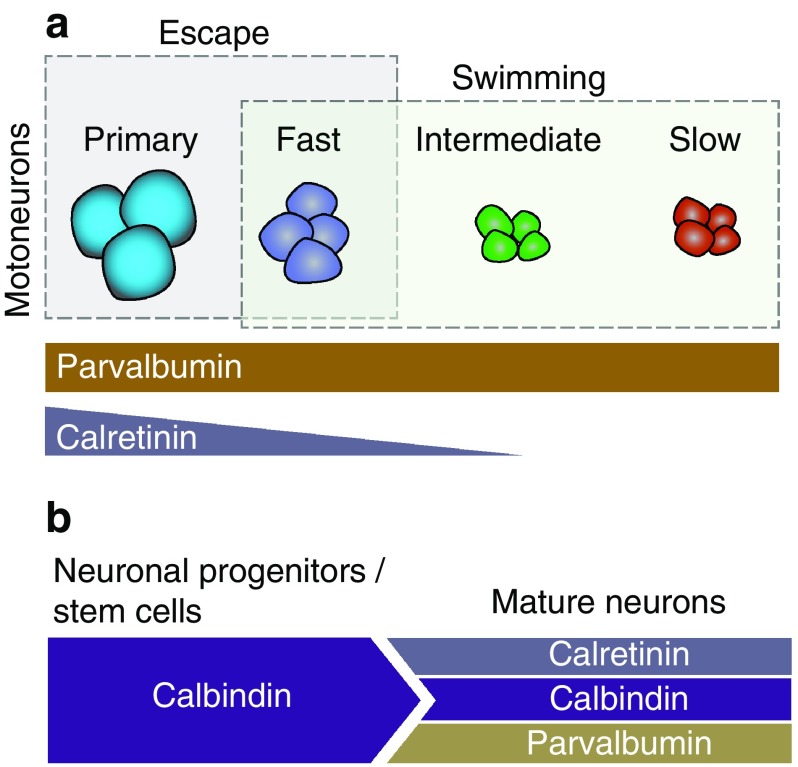
Schematic of the differential expression of the CBPs in the adult zebrafish spinal cord. **a** Motoneuron pools that contribute to the generation of fast movements (fast swimming and escape) utilize both CR and PV as calcium buffer proteins. **b** The progenitor cells/stem cells utilize CB to buffer their intracellular calcium. The mature neurons mainly use CR and PV as a calcium binding proteins. This suggests that CB can serve as anatomical marker to identify the developmental events that generate neurons in the adult zebrafish spinal cord

The initiation and generation of locomotion depend on dedicated neurons located in the spinal cord and supraspinal areas (Grillner 2003, 2006; Grillner and Jessell 2009; Esposito et al. 2014; Kiehn 2016; Goulding 2009). However, the identity of the neurons that form the locomotor networks that are sufficient to initiate and generate any locomotor activity, at different speeds and modalities, still remains unclear. Here, we utilize CBP expression in spinal and in supraspinal areas as a potential tool to characterize the locomotor network neuronal infrastructure. The present study is the first to directly classify the spinal cord neurons’ and the brain descending neurons’ diversity, distribution and morphology with respect to the calcium binding proteins CR, CB and PV in adult zebrafish. We show here that CBPs mark a highly heterogeneous population of neurons in the adult zebrafish spinal cord and in the reticulospinal areas. We also demonstrate that while most zebrafish axial motoneurons were labeled with PV, only motoneurons that generate the high speeds of swimming or participate in the escape response were in addition CR immunoreactive (Fig. 7a). In extrapolation, our data suggest that CR can be a potential valuable marker for the fast locomotor microcircuit module, possibly marking also the interneuron population which is part of this module. Finally, we show that CB immunoreactivity was mainly confined to a large population of cells surrounding the central canal, and we revealed that the vast majority of these cells were progenitor cells/stem cells (Fig. 7b). Hence, we suggest that new born neurons utilize CB as a regulator of their intracellular calcium and when they mature and start expressing a neurotransmitter phenotype the vast majority uses other calcium regulator proteins such as CR and PV (Fig. 7b).

In the vertebrate nervous system, the presence of calcium binding proteins is often associated with specific neurotransmitter phenotypes (Katsumaru et al. 1988; Celio 1990; Andressen et al. 1993). For instance, PV has been usually observed in GABAergic neurons located in the hippocampus, cerebellum and cortex (Katsumaru et al. 1988; Celio 1990; Andressen et al. 1993), whereas CR and CB can be associated with both excitatory and inhibitory neurons (Celio 1986; Aoki et al. 1990; Reynolds and Beasley 2001). Our analysis clearly indicates that there is no obvious correlation between the CBPs studied here and a given neurotransmitter in the adult zebrafish spinal cord.

Currently in the spinal cord the only cell type that has been strongly associated with different CBPs are Renshaw cells (Arvidsson et al. 1992; Carr et al. 1998; Sapir et al. 2004; Alvarez et al. 2005). Renshaw cells are inhibitory neurons deriving from the V1 population (Sapir et al. 2004; Alvarez et al. 2005) that release GABA or glycine to mediate recurrent inhibition to motoneurons (Cullheim and Kellerth 1981; Schneider and Fyffe 1992) and is well documented to contain CB and in a smaller degree also PV and CR (Sapir et al. 2004). Our findings demonstrate that a small population of neurons co-expresses CB and glycine in the adult zebrafish spinal cord. Although Renshaw cells have not been reported in the zebrafish spinal circuits, a previous study revealed that Engrailed-1 (a marker for V1 interneurons) is expressed in a small population of inhibitory glycinergic interneurons that possibly act in an analogous way to Renshaw cells in the mammalian spinal cord (Higashijima et al. 2004c). It is thus possible that these previously described Renshaw-like neurons in zebrafish contain also CB and constitute an evolutionary conserved population of neurons that later in the mammalian spinal cord forms the Renshaw cells, a question that should be addressed in further studies.

Several previous studies analyzed CBP expression in the vertebrate spinal cord (Fournet et al. 1986; Antal et al. 1990; Celio 1990; Ince et al. 1993; Ren et al. 1993; Sapir et al. 2004; Alvarez et al. 2005; Anelli and Heckman 2005; Morona et al. 2006a,b; Morona and González 2013) including fish (Maler et al. 1984; Denizot et al. 1988; Díaz-Regueira and Anadón 2000; Megías et al. 2003; Castro et al. 2005; Graña et al. 2013), however, many contradictory findings have been described about the presence of these proteins in spinal neuronal populations including motoneurons. In the adult zebrafish, the axial motoneurons form distinct pools related to the type of muscle fibers (slow, intermediate, fast) they innervate (Gabriel et al. 2011; Ampatzis et al. 2013). During swimming, different secondary motoneuron pools are sequentially recruited from slow, to intermediate, and finally to fast to cover the full range of locomotor speeds (Ampatzis et al. 2013). Moreover, the first developed primary motoneurons contribute only to the escape response (Ampatzis et al. 2013) and innervate fast muscle fibers. These findings suggest a differential contribution of axial motoneurons to the generation of locomotion at different speeds and modalities. In this study, we observed that both primary and secondary motoneurons strongly express PV, however, only the primary and some of the secondary fast motoneurons were found to contain CR, suggesting the functional significance of the presence of different CBPs in motoneurons to buffer intracellular calcium. In accordance with our results, vertebrate spinal cord motoneurons have been shown to contain CR (xenopus, Morona et al. 2006a, b, rats; lizard; Laslo et al. 2000; primates; Fahandejsaadi et al. 2004) including fish (grey mullet, Díaz-Regueira and Anadón 2000; zebrafish; Castro et al. 2005; lamprey; Megías et al. 2003; lungfish; Morona et al. 2010), however, mammalian spinal motoneurons have been shown to lack PV (Ince et al. 1993; Elliott and Snider 1995). The expression of CB appears to be more variable between different species. In our study, all axial motoneurons in the adult zebrafish spinal cord were found to be CB^−^. Similar to our observations the motoneurons of the turtle (Morona et al., 2007), and rat (Antal et al. 1990; Ren and Ruda 1994) were observed to lack CB. However, in lizards (Morona et al. 2006a), in primates (Fahandejsaadi et al. 2004), in xenopus (Morona et al. 2006b) and in other fish species several CB containing spinal motoneurons have been observed (Denizot et al. 1988; Díaz-Regueira and Anadón 2000; Megías et al. 2003; Morona et al. 2010). Besides this high variability regarding the presence of CBPs in secondary motoneurons, the prominent expression of CR in primary motoneurons that we observed has been documented also in other fishes (Denizot et al. 1988; Díaz-Regueira and Anadón 2000; Castro et al. 2005). Finally, we observed that zebrafish motoneurons contain PV, similar to previous studies that suggest the existence of PV in vertebrate brain motor nuclei and in the spinal cord (Philippe et al. 1993; Reiner et al. 1995; Sasaki et al. 2006).

The initiation of all animal body movements depends on the activation of brain descending neurons that project to the spinal cord (Grillner and Jessell 2009; Esposito et al. 2014; Kiehn 2016). The reticulospinal neurons drive the activity of the spinal locomotor networks that are responsible for the generation of locomotion at different speeds and modalities. Moreover, recent findings suggest that the supraspinal descending neurons provide in addition the necessary “stop” signals to terminate any ongoing movement (Bouvier et al. 2015). However, whether there are neuronal subpopulations that constitute functionally distinct modules in descending neurons remain unclear. Here we show that the vast majority of the brain descending neurons to spinal cord express PV and only a fraction of these neurons was found to express CR. Moreover, none of the reticulospinal neurons were observed to contain CB. The presence of CR in a large population of reticulospinal neurons was reported before in fish (Díaz-Regueira and Anadón 2000; Castro et al. 2005; Graña et al. 2012, 2013) as well as in other vertebrate species (Smeets and González 2000; Morona and González 2009). In contrast to previous studies demonstrating the existence of CB in brain to spinal cord descending neurons (Wang et al. 1996; Goodchild et al. 2000; Morona et al. 2006a,b), we could not reveal the presence of CB in retrogradely labeled zebrafish brain neurons. With regard to the presence of CBPs in the Mauthner cell, a gigantic reticulospinal neuron in the brainstem that generates the escape behavior (Zottoli 1977; Prugh et al. 1982), we found that these cells contain only PV. This observation is in agreement with observations in other teleosts, in which CR was not present in the Mauthner cell body (Crespo et al. 1998; Díaz-Regueira and Anadón 2000; Castro et al. 2005), whereas PV positive Mauthner cell bodies where identified before in tench (*Tinca tinca;* Crespo et al. 1998). Interestingly, the Mauthner cell axon in the spinal cord was found to lack PV. This is similar to results of previous studies that suggest the complementary expression of CBP in different cellular elements of the Mauthner cell, revealing the existence of a prominent complexity in the calcium buffering system (Crespo et al. 1998).

All three studied CBPs are known to participate in the regulation of intracellular calcium homeostasis, neurotransmitter release and synaptic alterations (Blaustein 1988; Miller 1991; Heizman and Braun 1992; Lledo et al. 1992; Andressen et al. 1993; Chard et al. 1993; Berridge et al. 2000). As such, Ca^2+^ regulators possess the ability to prevent or attenuate damage to cells due to toxicity that can be caused by the excessive entry of Ca^2+^ after prolonged neuronal activity (Scharfman and Schwartzkroin 1989). Such protection has been thought to underlie the selective survival, and conversely, selective vulnerability of neurons containing or lacking different CBPs (Morrison et al. 1998). Indeed, the differential expression or deficiency in CBPs in neurons has been suggested to be the key reason for the neuronal vulnerability to the progress of pathophysiological conditions associated with motoneuron degenerative diseases such as amyotrophic lateral sclerosis (ALS) (Ince et al. 1993; Alexianu et al. 1994; Elliott and Snider 1995; Reiner et al. 1995). It has been shown that already at presymptomatic stages of ALS, intracellular calcium levels in spinal motoneurons are increased (Siklos et al. 1998) and CBPs are practically absent (Alexianu et al. 1994; Elliot and Snider 1995; Ince et al. 1993; Reiner et al. 1995) indicating a neuroprotective role for CBPs (Mattson et al. 1991).

If the presence of CBPs could be indeed related to functional neuronal properties, then the anatomical distribution of these proteins holds a potentially exceptional tool for the study of the functional and anatomical organization of the spinal cord networks. More specifically, in mammals PV is often associated with fast spiking neurons in the hippocampus, in forebrain areas (Celio 1986; Kawaguchi 1993; Kawaguchi and Kubota 1993; Sik et al. 1995) and in the spinal cord (Solbach and Celio 1991). On the other hand, neurons related to sensory processing were shown to contain CR (Ren and Ruda 1994). Recent studies in the cerebellum of mice that lack CR or CB revealed altered firing patterns of granule cells (Gall et al. 2003; Cheron et al. 2004): CR-deficient granule cells exhibit faster action potentials and generate repetitive spike discharge. These results suggest that calcium binding proteins modulate neuronal excitability and activity of cerebellar circuits.

In the present study, we observed that cells surrounding the central canal express CB. We further saw that the vast majority of these cells (~ 80%) are neuronal progenitors/stem cells since they expressed the pluripotency marker Sox-2. From the remaining CB^+^ population, only a small fraction (~ 5%) expressed a neurotransmitter phenotype (Fig. 3b,e). It has been demonstrated that the expression of Sox-2 protein is not uniform (undetectable low-level protein expression) across the entire neural progenitor populations and that can explain the presence of CB^+^/Sox-2^−^ cells (Hutton and Pevny 2011; Hagey and Muhr 2014). In the adult zebrafish, as in all vertebrates, the proliferation niche of the spinal cord is situated around the central canal (Grandel et al. 2006; Kaslin et al. 2008; Hui et al. 2015). The main cell population in this area is the ependymal cells in both fish and mammals (Alfaro-Cervello et al. 2012; Hui et al. 2015). It is known that spinal neuronal precursors/stem cells exist within the population of central canal ependymal cells (Meletis et al. 2008). Under physiological conditions, ependymal cells self-renew and produce small numbers of glial progenitors that produce astrocytes and oligodendrocytes (Horner et al. 2000). However, in response to traumatic injury, ependymal cells increase their proliferative activity (Yamada et al. 1997) and act as neural stem cells to generate neuroblasts that proliferate and differentiate into neurons (Anderson et al. 1994; Meletis et al. 2008). In addition, mammalian spinal cord ependymal cells were found to contain CB (Ren and Ruda 1994; Zhang et al. 2016). Therefore, the results of the present study primarily indicate that in adult zebrafish the new born neurons that arise from the central canal ependymal cells use CB to buffer the intracellular calcium. However, once they mature and establish a neurotransmitter typology then the vast majority of these neuron uses CR and/or PV to mediate the calcium homeostasis.

## Abbreviations

5-HT: 5-hydroxytryptamine (serotonin)
CB: Calbindin D-28 k
CBPs: Calcium binding proteins
CC: Central canal
ChAT: Choline-acetyltransferase
CR: Calretinin
D: Dorsal
DON: Descending octaval nucleus
GABA: γ-Aminobutyric acid
Glut: Glutamate
IMRF: Intermediate reticular formation
IRF: Inferior reticular formation
MA: Mauthner cell axon
Mlf: Medial longitudinal fascicle
MN: Motoneuron
Nmlf: Nucleus of the medial longitudinal fascicle
P: Posterior
PV: Parvalbumin
RV: Rhombencephalic ventricle
SRF: Superior reticular formation
TeV: Tectal ventricle
V: Ventral
Va: Valvula cerebellum
